# Therapies crossing borders in European state socialism, 1950-1990. Introduction to the special issue

**DOI:** 10.1017/mdh.2025.10028

**Published:** 2025-10

**Authors:** Alexa Geisthövel, Daniela Koleva

**Affiliations:** 1 Institute for the History of Medicine, Charité Universitätsmedizin, Berlin, Germany; 2 Sofia University “St.Kliment Ohridski”, Department for History and Theory of Culture, Sofia, Bulgaria

**Keywords:** transnationalism, state socialism, medicine, health care, Bulgaria, GDR

## Abstract

The article introduces the special issue by mapping the field of pertinent scholarship and situating the articles with regard to the special angles and contributions they have to offer. As our five articles present case studies from Bulgaria and the GDR, both state socialist countries and their health care systems are portrayed here to provide context. The introduction locates each of the contributions and the overarching aims of the special issue within current scholarly discussions and demonstrates the issue’s innovative potential.

For several decades, social and historical sciences have depicted European state socialism as a monolithic bloc dominated by Soviet hegemony. Top-down command economy, party-states, an ideology of the collective, and repressive governance were distinguishing features of the so-called Second World, setting it clearly apart from its liberal capitalist competitor. With regard to science, technology, and biomedicine, European state-socialism, despite its self-proclaimed progressiveness, seemed to be cut off from international debates and lagging behind in development. In the last decade or so, as a result of what has been called the ‘cultural turn in Cold War studies’,^1^ the picture has been changing and has become more nuanced thanks to research that has steered away from the focus on nation-states and diplomacy, and from the Cold War reflex of either condemning or idealising. Instead, researchers have increasingly taken an interest in non-state actors and in smaller-scale processes of transfer, mediation, adaptation and (re)negotiation. As a consequence, it has been suggested that the impermeable Iron Curtain that divided Europe was in fact often a very permeable ‘Nylon Curtain’.^2^

## Cold-war medicine: detailing the picture

The field of medicine and health care was no exception to this. Quite the contrary: studies on medicine and science have very much contributed to this historiographical shift. Medical research and health care in state socialism were less prone to ‘securitisation’, in the sense of both political/ideological control, and tying them to state-security agendas. Hence, they were more open to international, namely ‘Western’ influences than other fields, especially those with higher ideological significance and those closer to the military industries, and to national security more broadly. Free accessible medical services were one of the main promises of socialism, so delivering those services had priority over ideological purity.^3^ Thus, medical technology and consumables crossed the Iron Curtain, as did drugs or endogenous substances, as in the case of Bulgarian pituitary glands for the production of growth hormone, as investigated by Daniela Koleva in this special issue.

Physicians and biomedical scientists were among those travelling experts who recent scholarship has identified as key actors in international collaboration and transnational entanglements.^4^ It has been established that some Eastern European protagonists, especially scientists and professionals, were well-connected with their peers around the globe, and kept up to date with the development of their fields. The fight against epidemic diseases and transplantation medicine is a case in point, as Milena Angelova and Alexa Geisthövel demonstrate in this issue.^5^ Also, psychoanalysis, supposedly suppressed in communist regimes for ideological reasons, actually resonated with Czech and Yugoslav therapists.^6^ In relation to the study of ageing and the history of gerontology and geriatrics, researchers have highlighted various types of synchronicities and entanglements.^7^ In general, mobile knowledge brokers with travel privileges were part of domestic power structures and represented their countries abroad. But they also had their own professional and individual agendas and were successfully striving for some degree of autonomy. As they managed to get access to knowledge and technical resources of their Western peers and were actively promoting international developments in their respective countries, they were able to exert considerable, albeit unacknowledged, influence on domestic policies and professional practice.^8^ Markus Wahl’s case study on the treatment of alcoholism corroborates this assumption in the field of health care.

However, looking at the Central and Eastern European (CEE) region as an illustration of the spread and impact of Western science across the Iron Curtain, or else to assess how CEE countries managed or failed in ‘catching up’, still privileges the Western perspective.^9^ Thus, a need has been felt for a more balanced historiography that describes parallels and differences in how Eastern and Western European countries dealt with challenges.^10^ A striking example is the pioneering studies on liberal economics, which showed how Eastern economists actively participated in international debates, made distinct contributions to global financial governance and brought about creative local adaptations.^11^ In the medical field, particularly, Dora Vargha in her path-breaking book on coping with the polio epidemics in 1950s Hungary has shown that specialised knowledge and technologies ebbed and flowed across borders ‘in ways that did not necessarily map onto the usual Cold War narratives’.^12^ Thus, the responses of former communist states to global health-related challenges do not always fit into neat divisions of ‘opening’ or ‘closing’. Competition and collaboration developed in parallel. In fact, sometimes competition occurred ‘*through* collaboration’.^13^

Moreover, this dynamic was characterised by the multiplication of non- and intergovernmental actors, such as the Red Cross and the World Health Organization (WHO) in the field of health care, as Milena Angelova shows in this issue, in her article on the attempts to control tuberculosis. The most prominent example of co-operation in international health campaigns remains the eradication of smallpox in the late 1960s and 1970s.^14^ At the same time, international health programmes were sites of another huge trend in the second half of the twentieth century, i.e. competing ‘globalisations’ in relations of both the First and the Second Worlds with the Global South. International medical encounters between the three worlds could take place on very different levels: in international organisations, such as WHO, in bilateral relationships founded on the principles of cultural cooperation or aid – such as building and staffing hospitals or training doctors from the Global South in CEE countries – in interinstitutional agreements between health care facilities and, last but not least, interpersonal networks.^15^

With the focus of Cold War historiography shifting to places, people, and events outside the US-Soviet dyad, attention has also been devoted to the smaller players and countries away from the central axis of the interbloc conflict.^16^ As a result, the ‘Soviet bloc’ has started to appear much less monolithic than before. Recent literature stresses the diversity of European state socialisms and puts under scrutiny the different paths that individual states took after the Stalinist period: from entrepreneurial self-management economies in Yugoslavia and Hungary to planned economy debt traps in Bulgaria; from mass civil society movements like Solidarność in Poland to tight control by the secret police in Romania and the GDR. Furthermore, socialist states, though under single-party rule, were not homogeneous entities, but like any other differentiated modern polities, they were composed of a multitude of agencies and actors with diverging, even contradicting agendas. That is, to judge what ‘East-Berlin’, ‘Sofia’, or ‘Moscow’ wanted or did would in many cases be too simplistic.

Our thematic issue aims to add to this strand of research, taking the discussion a step further. The authors see the Cold War as the broader context of the specific phenomena they seek to understand. In an attempt to steer away from ideologically grounded interpretations, we propose an approach based on ‘middle-range contextualisations’^17^, zooming in on the meso and even the micro level of institutions, regimes of knowledge production and therapeutic practices. Thus, we focus on European ‘East–West’ connections that were decisive for everyday medicine in terms of knowledge, infrastructures and practices. Our hypothesis is that the Cold War did not prevent some forms of internationalisation of biomedical research and therapies, and that these forms evolved with the changes of the societal, political, and economic circumstances.

Rather than following the usual US-Soviet axis or taking a global perspective, which has already been successfully attempted, we have chosen to focus on two ‘peripheral’ countries of the Eastern Bloc: the GDR and Bulgaria. We believe that this narrower focus will allow us to capture both the internal dynamics of the medical fields under scrutiny and their situatedness in their national and international contexts. Thus, new nuances can be added to classical topics such as Pavlovianism, as examined by Kristina Popova. The advantage of such an East-East comparison lies in ‘adding back the regional lens missing from transnational scholarship’, which recently has focused on West-Eastern and East-Southern entanglements.^18^ In addition, this is a way to tackle the issue of a certain ‘methodological nationalism’ pervading most of the research in the history of medicine in Eastern Europe.^19^

Although the structure of their economy was different, both the GDR and Bulgaria faced similar economic challenges.^20^ They were submissive Comecon members, enjoying little freedom in their economic policies (unlike more ‘rebellious’ members like Hungary). The instruments to pursue economic policies proved ineffective. In the GDR, the 1960s policy of foreign loans for reform programs and consumerism led to a permanent debt crisis since the 1970s, so in the 1980s, it constantly navigated on the verge of state bankruptcy.^21^ Bulgaria performed even worse. Between 1960 and 1990, Bulgaria defaulted three times on its government debt: in 1960 and 1976 on its debts to the Soviet Union, in 1990 (the effective default began in 1987) on the government debt to the so-called London Club of private creditors.^22^ The subsidisation of foreign debt obligations by Soviet supplies of energy resources was costly and hardly sustainable, and was increasing the dependency on the Soviet Union. By the late 1970s, the economic decline in both countries was already pronounced and unstoppable.

In both the GDR and Bulgaria, ideological control was relatively tight, especially in the first decades of the regime, and secret police surveillance was widely practised to control the movement of ideas, objects, and bodies. Moreover, the Bulgarian State Security and the East German Stasi seem to have collaborated throughout the whole period of the existence of their communist regimes.^23^ Yet another similarity between the GDR and Bulgaria was that, unlike Poland, Hungary and Czechoslovakia, dissident movements appeared relatively late and did not gain momentum until the late 1980s. It can be considered that intellectuals, including medical researchers, were co-opted by the regimes more successfully than in the mentioned CEE countries.

All in all, these similarities and interconnections between Bulgaria and the GDR make the study of the international professional connections and exchanges particularly illuminating. However, in other respects, the two countries represent varieties of European state socialism, which ensures added value to their comparison. Here, we are focusing on the differences in their health systems, which form the immediate settings of the case studies developed below.

## The GDR and Bulgaria: two medical worlds within European state socialism

Like the other countries of the Soviet bloc, the GDR and Bulgaria adopted from the Soviet Union the so-called Semashko model, whereby the healthcare system became an integral part of the planned economy.^24^ In the GDR, this included a revival of interwar schemes of socialised medicine.^25^ The common features of the model were state ownership of all medical facilities, salaried health workers, a high degree of governmental administration and chronic underfunding of healthcare. Still, each country adopted the model to a different extent, creating its version.

The GDR was a ‘welfare dictatorship’ (Konrad H. Jarausch) with highly developed medical care and strictly centralised health care administration, which, in the 1970s and 1980s, made the country an interesting partner for drug trials of Western pharmaceutical companies.^26^ In contrast with Bulgaria, in the aftermath of WWII, the medical profession was not cleansed. Specialists, even if they had a Nazi past, were direly needed, as were clinicians with a ‘bourgeois’ background, because many physicians fled to the FRG before and after 1961, as the formerly ‘free’ profession of private practice and self-organisation was put to an end in East Germany.^27^ Therefore, on the whole, the political control on appointments of qualified medical staff was insignificant while professional criteria were leading. Until the erection of the Berlin Wall, the professionals in East and West continued to meet at conferences or board meetings of associations and to publish in the established journals, so there was a shared professional public. Consequently, the Pavlovian doctrine imported from the Soviet Union never gained a strong foothold in the GDR.^28^ Even if the German-German bonds loosened and were cut off after 1961, there were still many connections, though it was much more complicated to get a travel permission for the FRG or West Berlin than for most other Western destinations.

As authorities considered health care an important field, and since many doctors fled the country, medicine was in the focus of the secret police. Though everyday medical practice was not very much politicised, three to five per cent of East German physicians reported to the secret police as unofficial informants, a distinctively higher percentage than in other professional groups. This could betray doctor-patient confidence, but mostly aimed at ‘securing’ the loyalty of health care personnel.^29^ Physicians involved in kidney replacement therapy, as portrayed by Alexa Geisthövel, also spied on colleagues at home and abroad, but just as often they used the conspirational setting for drawing attention to their work situation. Paradoxically, the informant-handler conversation proved to be a space for addressing deficiencies of the health care system in a relatively frank manner.

In the international arena, the GDR was marginalised after the geopolitical division of Germany. Gaining international recognition as an independent and legitimate state was at the core of East German diplomacy. This was achieved only in the early 1970s after the Basic Treaty with the FRG, which also meant that the GDR was finally accepted into the WHO in 1973. Thus, international contacts in medicine and health care always had the political angle of spreading word on the exemplary East German health care system, of proving competitiveness in the latest medical developments, not least as compared to East Germany’s Western rivalling sibling, the FRG.^30^ In the 1950s and early 1960s, the GDR actually could boast substantial achievements, for instance, in fighting polio, diphtheria, and infant mortality.^31^ Free access to state-of-the-art health care was realised in a tight network of outpatient treatment for curative and preventive care. For particular therapies, scarcity of means combined with hierarchical decision-making proved to be even advantageous for quality control and clinical outcomes.^32^ Initially, the GDR could also aspire to compete in prestigious treatments such as cancer therapy or organ transplantation, as Alexa Geisthövel shows in her case study. But even though considerable resources were directed into the treatment of terminal renal failure, the GDR fell behind the international development since the late 1970s. Thus, the fact that – due to the nature of organ replacement therapy in the 1960s – transnational exchange was inscribed into this prestigious project from the start, allowed East German transplantation medicine to integrate itself into the international peer community, but in the long run reinforced its peripheral position.

In a field of huge socio-political significance, we can see that the legalisation of first-trimester abortion in 1972 responded to West German developments, giving rise to intensified research on the ‘abortion pill’ as a tool for implementing the new national reproductive rights policy.^33^ Proving to be more invested in gender equality than the FRG, the topic of sexual hormones at the same time offered an opportunity to join the international research community. The GDR leadership welcomed that type of high-profile connections and had been eager to adopt WHO policies early on. This led to surprising side-effects, of which the risk factor concept is a good example: via risk thinking, so some scholars argue, elements of (neo)liberal governmentality were thriving in certain areas of 1970s/80s East German socialism, e.g. in psychological counselling or the management of cardiovascular diseases.^34^

On the other hand, several studies have shown that on an everyday level, medical practice was not fully controlled by the state or the leading professionals following along the official line. Flying under the radar, there was room for local initiatives that depended on committed individuals’ networks and choices.^35^ Markus Wahl’s article on alcoholism exemplifies how an officially taboo health issue was tackled by East German health care professionals. The treatment of alcoholism sought models elsewhere without the approval or support of the powerful health administration, as the issue at stake did not even exist officially. Physicians thus relied on several ‘foreign’ sources: disease concepts from the US and West Germany, therapeutic clubs from their Czech colleagues, but also from the internal other of state socialism, the Church. The example reveals the agency of local health care professionals, vis-à-vis official ignorance and a climate of moral condemnation.

Unlike the GDR, Bulgaria was in a catch-up regime, modernising its medicine and health care system. As the closest satellite of the Soviet Union, it not only faithfully adopted the Semashko model of health care,^36^ but its international collaboration in the field was, for a long time, oriented primarily towards Soviet medicine. Following the Expropriation Act of 23 December 1947, in two years, over 7000 companies – among them all private hospitals and clinics – were expropriated without compensation. Their owners were often banned from medical practice. Private consulting was discouraged, although it took a couple of decades to completely ban it in 1972.^37^

The situation of the medics in the immediate aftermath of the Second World War was different from that in the GDR, where a substantial continuity was preserved. In contrast, the medical profession in post-WWII Bulgaria experienced serious disruptions. Over eighteen per cent of c. 3,400 doctors active in 1944 in Bulgaria suffered political or administrative repressions in the following years.^38^ They were fired from their jobs, sentenced to imprisonment or interned in forced labour camps without sentences; some lost their lives. In the years following the Decree on the Purge of Teaching Staff (3 November 1944),^39^ forty-two professors were expelled from Sofia University, one-third of them being medics. Milder forms of repression, such as hampering careers, were more frequent but more difficult to document. In a series of purges among university students in the late 1940s and early 1950s, over 300 students of medicine were expelled from the university. (About half of them managed to restore their student status in later years, sometimes in a different degree programme, e.g. dentistry or veterinary medicine – a fact that points to the arbitrariness of the purges.) At the same time, participants in the communist resistance who were privileged to enrol at the university often did not have the qualities to become good doctors, teachers, or researchers. These ‘progressive’ students used to organise boycotts of the lectures of the ‘old-regime’ professors, often leading to their suspension from teaching. The massive repression of doctors (and intellectual elites in general) led to a decrease in the quality of medical education and research, especially in the first decade or so after the 1944 coup d’état.

Unlike the GDR, Bulgaria was probably the country where Sovietisation in all spheres, including health care and medical research, was most thoroughly carried out. First of all, as Milena Angelova demonstrates in this issue, based on the case of the BCG vaccines, existing and emerging collaborations with Western bodies (the Danish Red Cross in this case) were severed under Soviet pressure. Thus, bacteriological research and vaccination policies were charged with political overtones and instrumentalised for the legitimacy of the communist regime. A second layer of her study reveals how Bulgarian scientists, very much like their colleagues in the GDR, could manoeuvre between different loyalty requirements and pursue their research interests in the context of a new regime of knowledge production.

In parallel with the already mentioned implementation of the Semashko model of the organisation of health care, Marxist-Leninist epistemology was firmly established in all branches of medical education and research. In particular, the total imposition of I.P. Pavlov’s ideas and their ‘defence’ against ‘bourgeois distortions’ negatively affected many fields, from biology and physiology to psychiatry and education.^40^ The embrace of Soviet science went deeper to affect not only research but also therapeutic practices. As Popova demonstrates in this volume, after the infamous Pavlovian session (1951), clinical practice was steered toward conceptions that prioritised an organic-materialist approach acting upon the body to cure diseases that originated in the mind. Sleep therapy was a ‘Pavlovian’, thus ‘Soviet’ type of psychotherapy introduced in Bulgaria (and elsewhere) for psychiatric as well as internal ailments in the early 1950s. In a series of patient records, though, we can see that the ideological-scientific program did not smoothly translate into clinical routines, but met with reinterpretations and practical resistance. Thus, the well-known story of doctrinaire Pavlovianism is complicated and relativised based on the significance of local practices.

While Michurin and Lysenko were forgotten after the 1950s, university education in medicine bore the mark of the ideology till the very end of the regime. Instead of medical ethics, genetics and psychology, students had courses in dialectical and historical materialism, scientific communism and history of the Bulgarian communist party. To limit ourselves to one example, the world-renowned psychiatrist Nikola Shipkovenski published his book on psychiatric iatrogeny and ‘befreiende Psychotherapie’ (freeing psychotherapy) twice in German: in the GDR and Switzerland, so it was available to doctors in both Germanys, while it appeared in Bulgarian only in 2016.^41^ The idea that the physicians could (mostly unaware) iatrogenise their patients, causing such disorders as anxiety neurosis, hypochondria, neurasthenia and psychosomatic diseases, sometimes leading to suicide, did not match the materialist doctrine in its Marxist-Leninist version. Shipkovenski was subjected to political pressure in the late 1950s: he was criticised at two dedicated academic sessions for ignoring Pavlov’s teaching, insufficiently adhering to dialectical materialism and following ‘western science’.^42^

In the later decades, the ideological grip on medical sciences was loosened, and researchers could adhere to established academic norms. Shipkovenski and many of his colleagues did travel to international conferences, exchange ideas and research results with their colleagues and publish in international academic journals (with the clearance of a state-security officer who ensured that their articles did not contain any sensitive information). The more important aspect of the international context now was the economic asymmetry. In her article, Koleva explores how the need for foreign currency to provide research equipment and consumables determined the involvement of the Institute for Endocrinology, Gerontology and Geriatrics in Sofia in a very questionable exchange. As in the GDR, medical research and practice depended on Western imports, and institutions went to great lengths to secure them whenever possible.

Bringing together case studies from a range of medical fields in the two countries and exploring various forms of contact and circulation, the authors in this special issue aim to grasp both diversity within a medico-political system and the exchanges between different ones. They do not follow cosmopolitan individuals and their ‘transsystemic careers’, and they do not focus on particular events such as landmark international conferences or international professional associations in order to explore the transnational in the age of the Cold War.^43^ Instead, the articles focus on therapies from several different medical disciplines – from psychiatry to infectious diseases to hormone therapies and organ transplantation. International or national health policies, the treatment programmes and recommendations of medical disciplines were one thing, but their implementation into practice were another. While prevention and primary health care are arguably considered to have been at the core of ‘socialist’ health policies throughout the region, it is on the level of therapeutic practices (that could sometimes also be preventive) where bottom-up dynamics and medical – not political – initiative can be observed. Centring the analysis on therapies rather than policies, ideology, institutions, or professional careers provides an additional dimension to current scholarship, and focusing on cases allows for capturing complexities and ambivalences. Each article delves into the specificities of the respective case, moving forward national research on the topic of interest. But there are also complementarities and junctions among them, going beyond the methodological choice to challenge Cold-War binaries and focus on small-scale, localised situations. The articles are arranged in a loose chronological order, which superimposes the history of medicine and health care onto the history of state socialism in the two countries. Milena Angelova discusses an urgent challenge in the wake of the Second World War – tuberculosis – and the intervention of politics into medical research at the onset of the Cold War. Kristina Popova focuses on the Sovietisation of the early 1950s, explaining the impact of a combination of political and ideological factors on therapeutic practices through the so-called ‘sleep therapy’. Markus Wahl bridges the decades from the 1950s to the 1980s, showing how the ideological claim that addictions were alien to socialism gave way to local and expert initiatives to treat actual addictions, borrowing and adapting therapies developed in the West. Alexa Geisthövel broadens the focus from German-German exchange to wider international networks developing between the mid-1960s and 1980s to ensure the success of kidney transplantations. Finally, Daniela Koleva focuses on the last decade of the communist regime to explore an ambiguous way of coping with the chronic underfunding of medical research through the involvement of a Bulgarian institution in the international ‘bioeconomy’. Thus, taken together, the articles offer a broad overview of the evolution of socialist medicine: from Sovietisation and rigid ideological and political control (Popova, Angelova), through local and unofficial practical collaboration (Wahl), to institutionalised and contractual cooperation (Geisthövel, Koleva). As a whole, this special issue presents a variety of models of intra- and interbloc collaboration: some of them directly politically driven (Angelova), others circumventing political divides (Wahl, Koleva), and still others building stable international exchanges (Geisthövel).

As some of the articles in this issue demonstrate, the results often were far from the intentions, due to all kinds of systemic and contingent circumstances. Starting from concrete and circumscribed medical problems and local solutions to them, the authors analyse the scope and context of therapeutic transnationalism in order to get a more precise picture of where and when, for whom and in which direction, the Iron Curtain was permeable or not; if and how therapies changed when they crossed borders; how knowledge and practices circulated within the ‘bloc’ and across the Iron Curtain; and finally – how deep was the impact of ideology on such a purportedly humanist and universalist field as medicine.

## Footnotes

1

Robert Griffith , ‘The Cultural Turn in Cold War Studies’, Reviews in American History, 29, 1 (2001), 150–7.

2 This term was first suggested by György Péteri , ‘Nylon Curtain — Transnational and Transsystemic Tendencies in the Cultural Life of State-Socialist Russia and East-Central Europe’, Slavonica, 10, 2 (2004), 113–23, DOI: 10.1179/sla.2004.10.2.113 .

3

Dóra Vargha , Polio Across the Iron Curtain: Hungary’s Cold War with an Epidemic (Cambridge: Cambridge University Press, 2018), 97–100 33315356.

4 For other such actors see Simo Mikkonen and Pia Koivunen (eds.) Beyond the Divide: Entangled Histories of Cold War Europe (New York/Oxford: Berghahn, 2015).

5 See also Susan Gross Solomon , ‘East European Public Health and the Cold War: In Search of Circulation’, Revue d’Études Conparatives Est-Ouest, 49, 1 (2018), 7–43 .

6

Sarah Marks , ‘Suggestion, Persuasion and Work: Psychotherapies in Communist Europe’, European Journal of Psychotherapy & Counselling, 20, 1 (2018), 10–24 29527126 10.1080/13642537.2017.1421986PMC5827704; Ana Antic , Non-Aligned Psychiatry in the Cold War: Revolution, Emancipation and Re-Imagining the Human Psyche (Cham: Palgrave Macmillan, 2022); Mat Savelli , ‘“Peace and happiness await us”: Psychotherapy in Yugoslavia 1945–85’, History of the Human Sciences, 31, 4 (2018), 38–57 .

7

Daniela Koleva and Ignat Petrov , ‘Socialist Gerontology? Or Gerontology during Socialism? The Bulgarian Case’, History of the Human Sciences, 36, 3–4 (2023), 178–201 .

8

Dmitriy F. Chebotaryov , Vospominania (Kiyv: IPC ALKON NAN Ukrainy, 2008); Szabolcs László , ‘Trans-systemic Mobility, Travel Reports and Knowledge Acquisition in Cold War Hungary in the 1960s and 1970s’, European Review of History/Revue européenne d‘histoire, 30, 2 (2023), 204–33.

9

Adela Hîncu , ‘Introduction: “Peripheral Observations” and Their Observers’, in Adela Hîncu and Victor Karady (eds), Social Sciences in the Other Europe since 1945 (Budapest: Pasts Inc, Center for Historical Studies, Central European University, 2018), 1–25, here 2.

10

Esther Wahlen , ‘Turning Women into Alcoholics: The Politics of Alcohol in Late Socialist Czechoslovakia’, Heike Karge , Friederike Kind-Kovács , and Sara Bernasconi (eds), From the Midwife’s Bag to the Patient’s File: Public Health in Eastern and Southeastern Europe (Budapest and New York: Central European University Press, 2017), 243–62, here 245 .

11

Johanna Bockman and Gil Eyal , ‘Eastern Europe as a Laboratory for Economic Knowledge: The Transnational Roots of Neoliberalism’, American Journal of Sociology, 108, 2 (2002), 310–52, doi:10.1086/344411 .

12 Vargha, *op. cit.* (note 3), 53.

13

Johanna Folland , ‘“Not even the Highest Wall can Stop AIDS”: Expertise and Viral Politics at the German-German Border’, Central European History, 56, 2 (2023), 255–69, here 258. Emphasis in the original.

14

Erez Manela , ‘Smallpox and the Globalization of Development,’ in Stephen Macekura and Erez Manela (eds), The Development Century: A Global History (Cambridge and New York: Cambridge University Press, 2018), 83–104 .

15

Iris Borowy , ‘Medical Aid, Repression, and International Relations: The East German Hospital at Metema’, Journal of the History of Medicine and Allied Sciences, 71, 1 (2015), 64–92 26037639 10.1093/jhmas/jrv010; Gross Solomon, *op. cit.* (note 5); Bogdan C. Iacob et al., ‘State Socialist Experts in Transnational Perspective. East European Circulation of Knowledge during the Cold War (1950s–1980s): Introduction to the Thematic Issue’, East Central Europe 45, 2–3 (2018), 145–59; Dora Vargha, ‘Technical assistance and socialist international health: Hungary, the WHO and the Korean War’, *History and Technology*, *36*, 3–4 (2020), 400–17; Bogdan C. Iacob *, ‘* Health’ in James Mark and Paul Betts (eds) Socialism Goes Global: The Soviet Union and Eastern Europe in the Age of Decolonization (Oxford: Oxford University Press, 2022), 255–89; Bogdan C. Iacob , ‘Malariology and decolonization: Eastern European experts from the League of Nations to the World Health Organisation’, Journal of Global History, 17, 2 (2022), 233–53; Walter Bruchhausen and Iris Borowy , ‘Primary Health Care and Foreign Aid: A Tale of Two Germanys’, Journal of the History of Medicine and Allied Sciences, 79, 1 (2024), 65–89 37364178 10.1093/jhmas/jrad034; Michitake Aso , ‘Performing National Independence through Medical Diplomacy: Tuberculosis Control and Socialist Internationalism in Cold War Vietnam’, The British Journal for the History of Science, 57, 2 (2024), 205–2010.1017/S000708742400048739429122; Paula A. Michaelsa and Stéphanie Panichelli-Batall , ‘An Unbreakable Friendship: Soviet Medical Internationalism in Cuba in the 1960s’, History Australia 21, 4 (2024), 642–60; Mila Maeva, ‘Bulgarian medical personnel abroad as privileged persons during state socialism’, *Proceedings from the 12^th^ Joint Conference of Bulgarian and North American Scholars “Living Bridges Across Time”* (forthcoming).

16

Theodora Dragostinova , The Cold War from the Margins: A Small Socialist State on the Global Cultural Scene (Ithaca: Cornell University Press, 2021).

17

Joel Isaac , ‘The Human Sciences in Cold War America’, Historical Journal, 50, 3 (2007), 725–46.

18

Adela Hîncu and Agata Zysiak , ‘Socialist Culture, Participation and Expert Knowledge in Poland and Romania in the Long 1960s’, European Review of History/Revue européenne d‘histoire, 30, 2 (2023), 234–56, here 235–6.

19 For an excellent overview see Marius Turda , ‘History of Medicine in Eastern Europe, Including Russia’, in Mark Jackson (ed.), Oxford Handbook of the History of Medicine (Oxford: Oxford University Press, 2011), 96–117 .

20 Many of these challenges were the same for the whole CEE region, such as the growth of GDP per capita, which was significantly lower than that in Western European countries, especially from the 1970s on: https://www.statista.com/statistics/1073158/europe-gdp-per-capita-by-region-1950-1998/.

21

André Steiner , ‘The Decline of Soviet-Type Economies’, in Juliane Fürst , Silvio Pons and Mark Selden (eds), The Cambridge History of Communism. Volume III: Endgames? Late Communism in Global Perspective, 1968 to the Present (Cambridge: Cambridge University Press, 2017), 203–23.

22 Daniel Vachkov and Martin Ivanov, *Bulgarskiat vynshen dylg 1944-1989. Bankrutyt na komunisticheskata ikonomika* [Bulgarian foreign debt 1944-1989. The bankruptcy of the communist economy] (Sofia: Institute for the Study of the Recent Past/Ciela, 2008).

23 See the collection of documents published by the Bulgarian Committee for Disclosing the Documents and Announcing Affiliation of Bulgarian Citizens to the State Security and the intelligence Services of the Bulgarian National Army (CDDAABCSSISBNA) known as ‘ComDos’: *State Security and the STASI.* Sofia: CDDAABCSSISBNA (2014).

24 Named after Nikolai Semashko (1874-1949), the first head of the Narkomzdrav (People’s Commissariat of Public Health) based on the principle of universally accessible, free of charge health care provided by the state through a centralised health administration. While effective at its start due to the pooling of funds and the extension of coverage, it later exhibited the drawbacks of state monopoly and heavy bureaucracy. See Milena Angelova , ‘Vizionerstvo i zdrave: modelat “Semashko” i savetizatsiata na obshtestvenoto zdraveopazvane v Bulgaria (1944-1951)’, Balkanistic Forum, 3 (2021), 74–103, DOI: 10.37708/bf.swu.v30i3.4 ; Andreas Heinrich , ‘The Emergence of the Socialist Healthcare Model After the First World War’, in Frank Nullmeier , Delia Gonzalez de Reufels and Herbert Obinger (eds), International Impacts on Social Policy: Short Histories in Global Perspective (Cham: Palgrave Macmillan, 2022), 35–46 .

25 Udo Schagen and Sabine Schleiermacher, ‘Gesundheitswesen und Sicherung bei Krankheit’ in: Dierk Hoffmann and Michael Schwartz (eds), *DDR 1949-1961. Im Zeichen des Aufbaus des Sozialismus* (= Geschichte der Sozialpolitik in Deutschland seit 1945, vol. 8) (Baden-Baden: Nomos 2004), 390–433.

26

Volker Hess , Laura Hottenrott , and Peter Steinkamp , Testen im Osten: DDR-Arzneimittelstudien im Auftrag der westlichen Pharmaindustrie, 1964-1990 (Berlin: Be.Bra Wissenschaft Verlag, 2016).

27

Anna-Sabine Ernst , ‘Die beste Prophylaxe ist der Sozialismus’: Ärzte und Hochschullehrer in der SBZ/DDR 1945–1961 (Münster: Waxmann, 1996); Markus Wahl , Medical memories and experiences in Postwar East Germany (London and New York: Routledge Taylor & Francis Group, 2019), ch. 1.

28 Ernst, *op. cit.* (note 27), 308–32; Steffen Dörre , Zwischen NS-‘Euthanasie’ und Reformaufbruch. Die psychiatrischen Fachgesellschaften im geteilten Deutschland (Berlin: Springer, 2021), 315–28.

29

Francesca Weil , Zielgruppe Ärzteschaft: Ärzte als inoffizielle Mitarbeiter des Ministeriums für Staatssicherheit der DDR (Göttingen: V & R unipress, 2008); Jutta Begenau , Staatssicherheit an der Charité: der IM ‘Harald Schmidt’ und die ‘Sicherheitspolitik von Partei und Regierung’ an der Charité 1972-1987 (Berlin: Metropol, 2017).

30

Melanie Arndt , Gesundheitspolitik im geteilten Berlin, 1948 bis 1961 (Köln: Böhlau, 2009).

31

Anette Hinz-Wessels , ‘Medizinische Verflechtung und Systemkonkurrenz im Kalten Krieg: Poliobekämpfung im geteilten Berlin’, Medizinhistorisches Journal, 55, 2 (2020), 132–71.

32

Thomas Schlich , ‘Degrees of Control: The Spread of Operative Fracture Treatment with Metal Implants. A Comparative Perspective on Switzerland, East Germany and the USA, 1950s-1990s’ in: Jennifer Stanton (ed.) Innovations in Health and Medicine. Diffusion and Resistance in the Twentieth Century (London and New York: Routledge 2002), 106–25, here 108–112.

33

Donna Harsch , ‘Society, the State and Abortion in East Germany 1950-1972’, The American Historical Review, 102, 1 (1997), 53–84 11623498 . For the GDR as an international testing ground for sexual hormones see Laura Hottenrott’s research project https://medizingeschichte.charite.de/forschung/taming_the_european_leviathan/socialist_testing_grounds (accessed 28 February 2025).

34

Stefan Offermann , ‘Socialist Responsibilization: the Government of Risk Factors for Cardiovascular Diseases in the German Democratic Republic in the 1970s’, Rethinking History, 23, 2 (2019), 210–32; Viola Balz , ‘Riskante Selbstverhältnisse. Alkohol im Spiegel des Gesundheitsfilms in der DDR’, Viola Balz and Lisa Malich (eds), Psychologie und Kritik: Formen der Psychologisierung nach 1945 (Wiesbaden: Springer, 2020), 165–86.

35

Michael Geyer , ‘Wie frei war ich als Psychotherapeut in der “Diktatur des Proletariats”?’, in Bernhard Strauß , Rainer Erices , Susanne Guski-Leinwand , and Ekkehardt Kumbier (eds), Seelenarbeit im Sozialismus. Psychologie, Psychiatrie und Psychotherapie in der DDR (Gießen: Psychosozial-Verlag, 2022), 183–209 .

36 For details, see Angelova, *op. cit.* (note 24).

37 Ordinance of the State Council of 18 July 1972.

38 Kiril Milenkov (ed.), *Bulgarski lekari i studenti po medicina – zhertvi na komunisticheskia terror 1944-1989* [Bulgarian doctors and students of medicine – victims of the communist terror] (Sofia: Bulgarski lekarski sayuz, 2003). Available online: https://www.decommunization.org/Communism/Bulgaria/Lekari.htm

39 Naredba zakon za prochistvane na uchitelskia i prepodavatelskia personal v narodnite osnovni I sredni uchilishta, uchitelski instituti i Universiteta i visshite uchilishta i akademii, *Darzhaven vestnik* [State Gazette] N 243 / 03.11.1944.

40 Koleva and Petrov, *op. cit.* (note 7); Kristina Popova , ‘Visshata nervna deinost na socializma. Revolutsionnoto prilozhenie na edna kucheshka slyunka v preduchilishtnoto vazpitanie prez 50-te godini’ in Daniela Koleva (ed.), Tyaloto pri Socializma: Obraztsi, obrazi, tabuta (Sofia: Riva, 2016), 19–56 and this volume.

41

Nikola Schipkowensky , Iatrogenie oder befreiende Psychotherapie: Der psychische Einfluss des Arztes auf seine Patienten (Leipzig: S. Hirzel, 1965); Nikola Schipkowensky , Iatrogenie oder befreiende Psychotherapie: Der psychische Einfluss des Arztes auf seine Patienten (Basel: Schwabe Verlag, 1977); Nikola Shipkovenski , Iatrogenia ili osvobozhdavashta psihoterapia (Sofia: Iztok-Zapad, 2016).

42 Such sessions targeting certain scholars were quite common in the 1950s’ Bulgarian academia. See Koleva and Petrov, *op. cit.* (note 7), 181–2 for the session denouncing Dragomir Mateev, professor of physiology.

43

György Péteri , ‘Alexander Szalai: A Transsystemic Career and Hungarian Sociology in the Cold War Era’, East Central Europe 50, 2–3 (2023), 328–79.

